# Effects of *C-phycocyanin* and *Spirulina* on Salicylate-Induced Tinnitus, Expression of NMDA Receptor and Inflammatory Genes

**DOI:** 10.1371/journal.pone.0058215

**Published:** 2013-03-22

**Authors:** Juen-Haur Hwang, Jin-Cherng Chen, Yin-Ching Chan

**Affiliations:** 1 Department of Otolaryngology, Buddhist Dalin Tzu-Chi General Hospital, Dalin, Chiayi, Taiwan; 2 School of Medicine, Tzu Chi University, Hualien, Taiwan; 3 Department of Neurosurgery, Buddhist Dalin Tzu-Chi General Hospital, Dalin, Chiayi, Taiwan; 4 Department of Food and Nutrition, Providence University, Shalu, Taichung, Taiwan; University of Florida, United States of America

## Abstract

Effects of *C-phycocyanin* (*C-PC)*, the active component of *Spirulina platensis* water extract on the expressions of *N*-methyl *D*-aspartate receptor subunit 2B (NR2B), tumor necrosis factor–α (TNF-α), interleukin-1β (IL-1β), and cyclooxygenase type 2 (COX-2) genes in the cochlea and inferior colliculus (IC) of mice were evaluated after tinnitus was induced by intraperitoneal injection of salicylate. The results showed that 4-day salicylate treatment (unlike 4-day saline treatment) caused a significant increase in NR2B, TNF-α, and IL-1β mRNAs expression in the cochlea and IC. On the other hand, dietary supplementation with *C-PC* or *Spirulina platensis* water extract significantly reduced the salicylate-induced tinnitus and down-regulated the mRNAs expression of NR2B, TNF-α, IL-1β mRNAs, and COX-2 genes in the cochlea and IC of mice. The changes of protein expression levels were generally correlated with those of mRNAs expression levels in the IC for above genes.

## Introduction

Tinnitus can be perceived in one or both ears or in the head in the absence of acoustic stimulation. The prevalence of chronic tinnitus is estimated between 10.1% to 14.5% in adult population [1], and increased with age [2]. Salicylate-induced tinnitus in mice has been a popular animal model for the study of tinnitus [3]. High doses of salicylate (250–300 mg/kg sodium Salicylate, i.p.) are known to reduce otoacoustic emissions, elevate hearing thresholds, and reliably induce tinnitus [3]–[6].

Some mechanisms were proposed to explain the causes of tinnitus. For example, tinnitus may arise from an increase in excitatory neurotransmission, and was associated with *N*-methyl *D*-aspartate receptor (NMDA receptor, NR) activity [4]. Recently, our study group found that mRNA expression levels of the NR subtype 2B (NR2B) gene, tumor necrosis factor –α (TNF-α) and interleukine-1β (IL-1β) genes were elevated significantly in the cochlea and in the inferior colliculus (IC) in salicylate-induced tinnitus [5], [6]. And, we suggested that the proinflammatory cytokines might lead to tinnitus directly or via modulating NR gene expression [5], [6].

Although some medications appeared to be effective for tinnitus, the results were still not so satisfactory and/or their adverse side effects prevent them as a regular treatment for mice and/or humans [7]–[9]. Therefore, it is still worth searching for more “safe and effective” medications for tinnitus.

*Spirulina* is a microscopic blue-green algae living both in sea and fresh water. It is composed of high quality protein, iron, gamma-linolenic fatty acid, carotenoids, vitamins B_1_ and B_2_, minerals, and its active component: C-phycocyanin(*C-PC)*, etc [10]. It has been reported that *Spirulina platensis* water extract or *C-PC* exerts anti-oxidative, anti-inflammatory activities and neuroprotective effects via inhibition of COX [11] and/or nicotinamide adenine dinucleotide phosphate (NADPH) oxidase enzymes [12]. Meanwhile, as we mentioned above, tinnitus were associated with up-regulation of the NR2B and proinflammatory genes [5], [6]. So, *Spirulina* might be also a good candidate for prevention or treatment of tinnitus.

In this study, we aim to investigated whether *C-PC* or *spirulina platensis* water extract could reduce the tinnitus score and expression levels of NR2B, TNF-α, IL-1β, and COX-2 genes in the cochlea and IC in response to intraperitoneal injections of salicylate.

## Results

**Figure 1:**
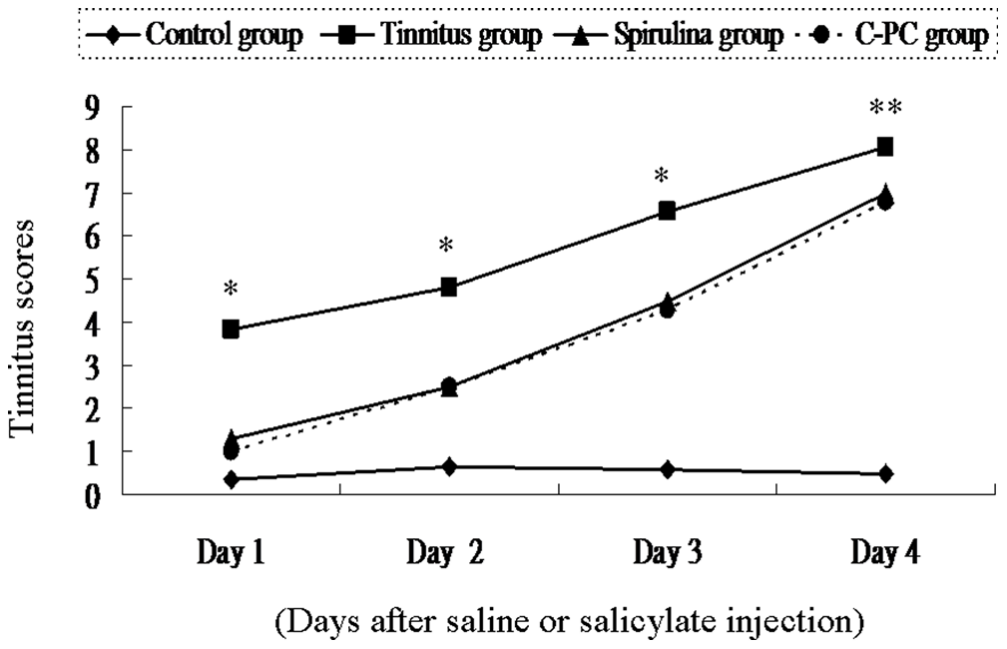
The tinnitus scores of four groups after intraperitoneal saline or salicylate injection. The tinnitus scores were significantly higher in the salicylate group than the control group on each day. In addition, the tinnitus scores of the *Spirulina* group and *C-PC* group were significantly lower than those of the salicylate group on each day (*p<0.001 at days 1, 2, and 3 for both groups; **p = 0.037 and 0.005 on day 4, respectively).

Figure 1 showed the tinnitus score was elevated day-by-day after intraperitoneal injection of 300 mg/kg sodium salicylate (tinnitus group) but not after injection of saline (control group). The mean tinnitus score at day 4 was 0.5±0.5 for the control group, 8.0±1.5 for the salicylate group, 7.0±1.3 for the *Spirulina* group, and 6.8±1.1 for the *C-PC* group. The differences in tinnitus score among the four groups were significant on each day (one-way ANOVA, p<0.0001). Post-hoc analysis showed that the tinnitus scores of the *Spirulina* group and *C-PC* group were significantly lower than those of the salicylate (tinnitus) group on each day (p<0.001 at days 1, 2, and 3 for both groups; p = 0.037 and 0.005 on day 4, respectively).

**Figure 2:**
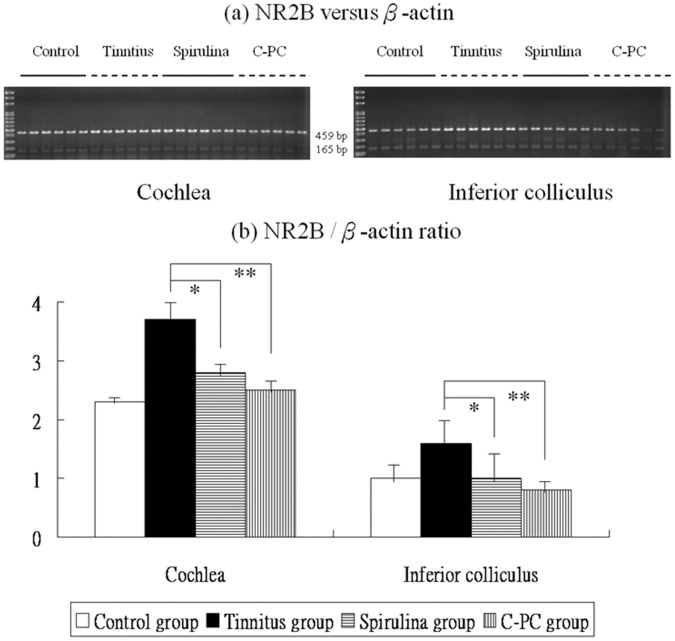
The levels of NR2B mRNA expression in the four groups (a,b). There are significant differences in these levels among the four groups. The NR2B mRNA expression were significantly higher in the salicylate group than the control group. Compared to the tinnitus group, the *Spirulina* group (*) or *C-PC* group (**) exhibits significantly reduced NR2B mRNA levels in the cochlea and IC.

**Figure 3:**
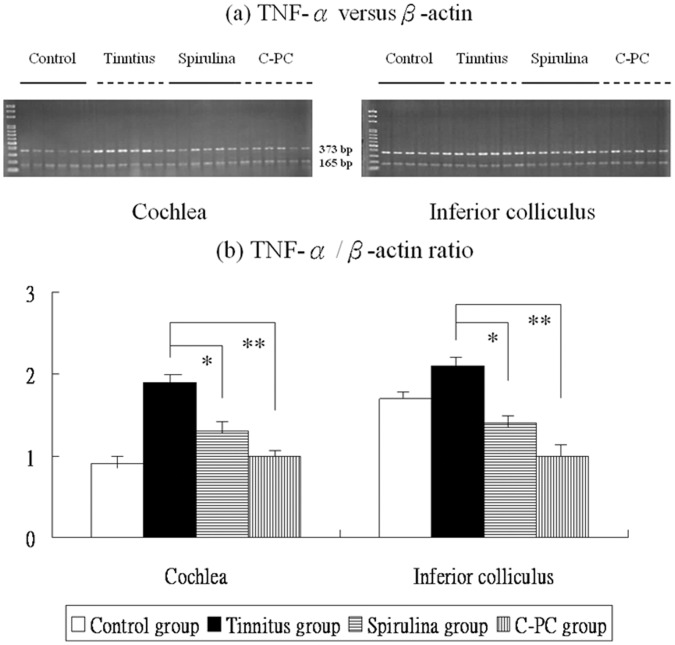
The levels of TNF-α mRNA expression in the four groups (a,b). There are significant differences in these levels among the four groups. The TNF-α mRNA expression were significantly higher in the salicylate group than the control group. Compared to the tinnitus group, the *Spirulina* group (*) or *C-PC* group (**) exhibits significantly reduced TNF-α mRNA levels in the cochlea and IC.

**Figure 4:**
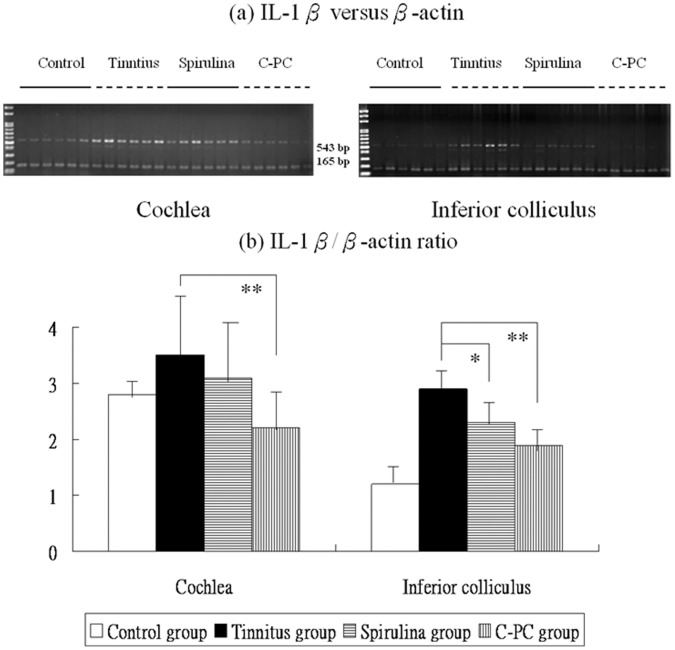
The levels of IL-1β mRNA expression in the four groups (a,b). There are significant differences in these levels among four groups. The IL-1β mRNA expression were significantly higher in the salicylate group than the control group. Compared to the tinnitus group, the *Spirulina* group (*) exhibits significantly reduced IL-1β mRNA level in the IC, whereas the *C-PC* group (**) exhibits significantly reduced IL-1β mRNA level in the cochlea and IC.

**Figure 5:**
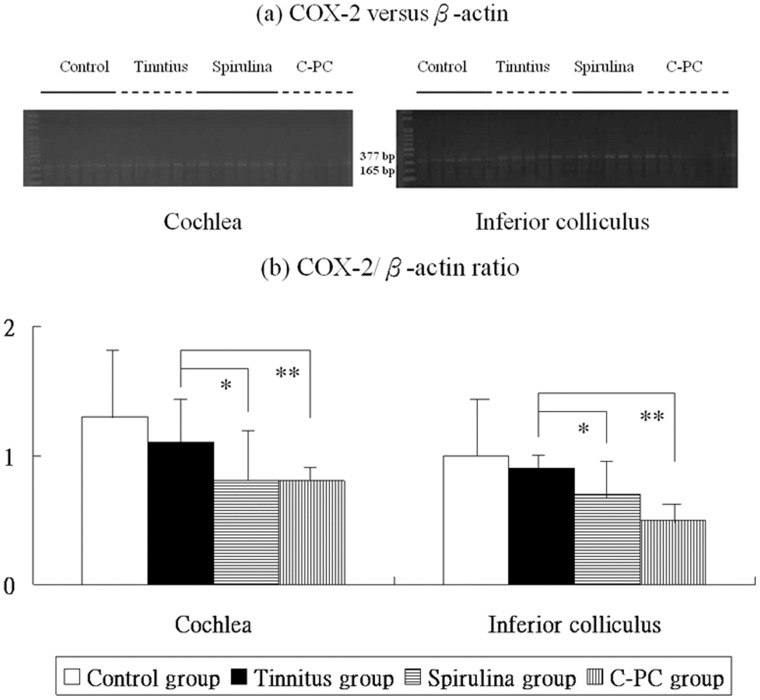
The levels of COX-2 mRNA expression in the four groups (a,b). There are significant differences in these levels among the four groups. The COX-2 mRNA expression were significantly higher in the salicylate group than the control group. Compared to the tinnitus group, the *Spirulina* group (*) or *C-PC* group (**) exhibits significantly reduced COX-2 mRNA levels in the cochlea and IC.

Figures 2, 3, 4 and 5 showed the mRNA expression levels of NR2B, TNF-α, IL-1β, and COX-2 in the four groups. The respective differences in NR2B, TNF-α, IL-1β, and COX-2 mRNA level among the four groups were significant (one-way ANOVA, p<0.001).

Post-hoc analysis showed that, compared to the control group, the tinnitus group had significantly increased NR2B mRNA levels in the cochlea (3.7±0.5 *versus* 2.3±0.1, p<0.001) and IC (1.6±0.6 *versus* 1.0±0.4, p = 0.003). However, NR2B mRNA levels were significantly decreased in the cochlea (2.8±0.3 *versus* 3.7±0.5, p<0.001) and IC (1.0±0.7 *versus* 1.6±0.6, p = 0.001) of the *Spirulina* group and the *C-PC* group (cochlea: 2.5±0.3 *versus* 3.7±0.5, p<0.001; IC: 0.8±0.2 *versus* 1.6±0.6, p<0.001) compared to the tinnitus group (Figure 2).

Post-hoc analysis showed that, compared to the control group, the tinnitus group had significantly increased TNF-α mRNA levels in the cochlea (1.9±0.2 *versus* 0.9±0.1, p<0.001) and IC (2.1±0.2 *versus* 1.7±0.2, p<0.001). However, TNF-α mRNA levels were significantly decreased in the cochlea (1.3±0.1 *versus* 1.9±0.2, p<0.001) and IC (1.4±0.2 *versus* 2.1±0.2, p<0.001) of the *Spirulina* group and *C-PC* group (cochlea: 1.0±0.1 *versus* 1.9±0.2, p<0.001; IC: 1.0±0.1 *versus* 2.1±0.2, p<0.001), compared with the tinnitus group (Figure 3).

Post-hoc analysis showed that, compared to the control group, the tinnitus group had significantly increased the IL-1β mRNA levels in the cochlea (3.5±1.1 *versus* 2.8±0.3, p = 0.031) and IC (2.9±0.5 *versus* 1.2±0.5, p<0.001). However, IL-1β mRNA level was significantly decreased in the IC (2.3±0.7 *versus* 2.9±0.5, p = 0.002) but not in the cochlea (3.1±1.1 *versus* 3.5±1.1, p = 0.473) of the *Spirulina* group, and significantly decreased in the cochlea (2.2±0.7 *versus* 3.5±1.1, p<0.001) and IC (1.9±0.6 *versus* 2.9±0.5, p<0.001) of the *C-PC* group (Figure 4).

Post-hoc analysis showed that, compared to the control group, the tinnitus group had similar COX-2 mRNA level in the cochlea (1.1±0.3 *versus* 1.3±0.5, p = 0.205) and IC (0.9±0.1 *versus* 1.0±0.4, p = 0.188). Nonetheless, COX-2 mRNA level was significantly decreased in the cochlea (0.8±0.4 *versus* 1.1±0.3, p = 0.034) and IC (0.7±0.3 *versus* 0.9±0.1, p = 0.021) of the *Spirulina* group and *C-PC* group (cochlear: 0.8±0.1 *versus* 1.1±0.3, p = 0.009; IC: 0.5±0.2 *versus* 0.9±0.1, p<0.001) compared with the tinnitus group (Figure 5).

**Figure 6:**
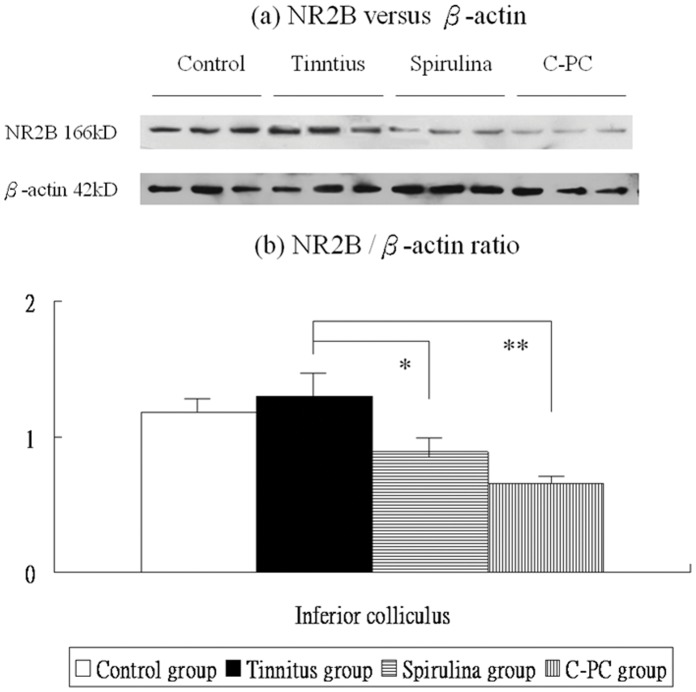
The levels of NR2B protein expression in the IC (a,b). There are significant differences in these levels among the four groups. The NR2B mRNA expression were not significantly higher in the salicylate group than the control group. Compared to the tinnitus group, the *Spirulina* group (*) or *C-PC* group (**) exhibits significantly reduced NR2B protein levels in the IC.

**Figure 7:**
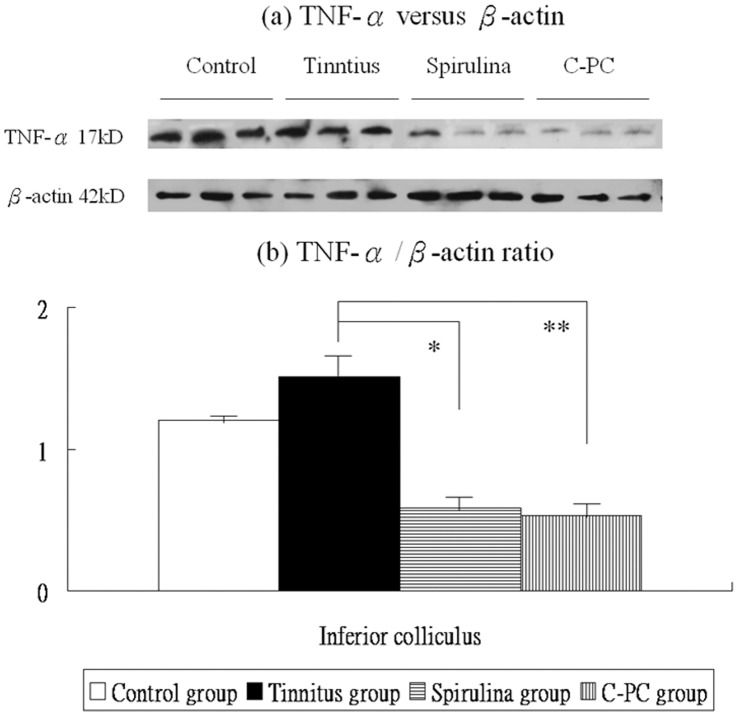
The levels of TNF-α protein expression in the IC (a,b). There are significant differences in these levels among the four groups. The TNF-α mRNA expression were not significantly higher in the salicylate group than the control group. Compared to the tinnitus group, the *Spirulina* group (*) or *C-PC* group (**) exhibits significantly reduced TNF-α protein levels in the IC.

**Figure 8:**
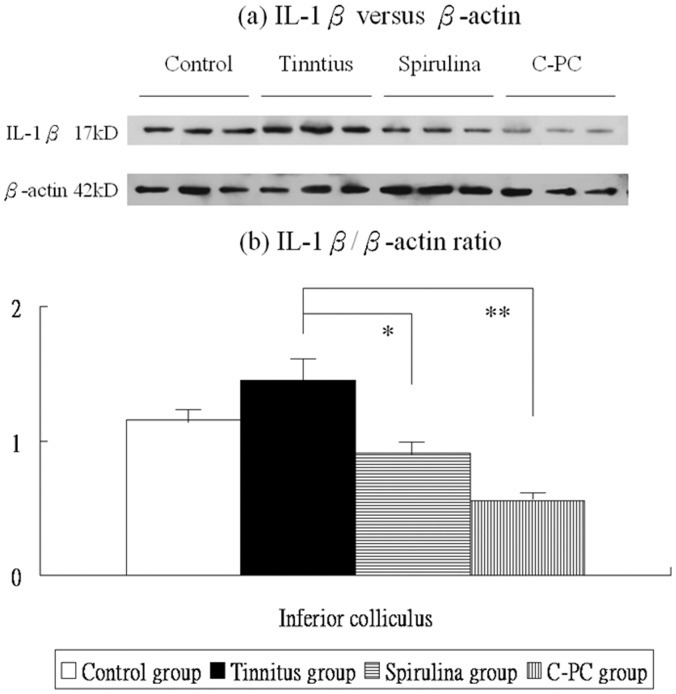
The levels of IL-1β protein expression in the IC (a,b). There are significant differences in these levels among four groups. The IL-1β protein expression were not significantly higher in the salicylate group than the control group. Compared to the tinnitus group, the *Spirulina* group (*) or *C-PC* group (**) exhibits significantly reduced TNF-α protein levels in the IC.

**Figure 9:**
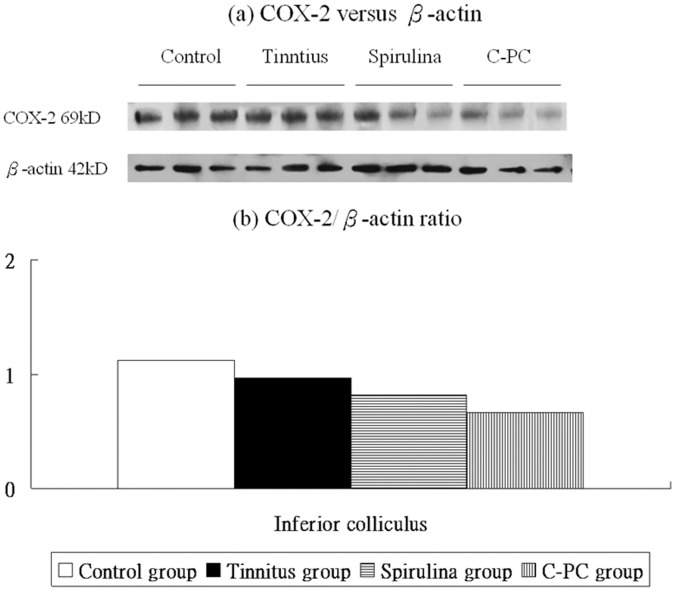
The levels of COX-2 protein expression in the IC (a,b). There are not significant differences in these levels among the four groups.

Figure 6, 7, 8 and 9 showed the protein expression levels of NR2B, TNF-α, IL-1β, and COX-2 in the IC. The respective differences in NR2B, TNF-α, and IL-1β protein level (one-way ANOVA, p<0.001) among the four groups were significant, but not in COX-2 (one-way ANOVA, p = 0.056).

Post-hoc analysis showed that, compared to the control group, the tinnitus group had not significantly increased NR2B protein levels in the IC (1.30±0.17 *versus* 1.18±0.12, p = 1.000). However, NR2B protein levels in the IC were significantly decreased in the *Spirulina* group (0.89±0.08 *versus* 1.30±0.17, p = 0.014) and in the *C-PC* group (0.66±0.04 *versus* 1.30±0.17, p = 0.001), compared with the tinnitus group (Figure 6).

Post-hoc analysis showed that, compared to the control group, the tinnitus group had not significantly increased TNF-α protein levels in the IC (1.51±0.19 *versus* 1.20±0.02, p = 0.085). However, TNF-α protein levels in the IC were significantly decreased in the *Spirulina* group (0.59±0.13 *versus* 1.51±0.19, p<0.001) and in the *C-PC* group (0.53±0.04 *versus* 1.51±0.19, p<0.001), compared with the tinnitus group (Figure 7).

Post-hoc analysis showed that, compared to the control group, the tinnitus group had not significantly increased IL-1β protein levels in the IC (1.45±0.18 *versus* 1.16±0.08, p = 0.057). However, IL-1β protein levels in the IC were significantly decreased in the *Spirulina* group (0.91±0.07 *versus* 1.45±0.18, p = 0.001) and in the *C-PC* group (0.56±0.01 *versus* 1.45±0.18, p<0.001), compared with the tinnitus group (Figure 8).

Compared to the control group (1.12±0.10), the tinnitus group (0.97±0.17, p = 1.000), *Spirulina* group (0.82±0.08, p = 0.401), and *C-PC* group (0.66±0.28, p = 0.071) had decreased COX-2 protein levels in the IC, but the differences were not significant (Figure 9).

## Discussion

This experimental study showed that the both of *spirulina platensis* water extract and its active component (*C-PC*) could reduce salicylate-induced tinnitus and reduce expression of NR2B, TNF-α, IL-1β, and COX-2 genes in the cochlea and IC. As we mentioned above, salicylate-induced tinnitus was associated with up-expression of NR2B, TNF-α, and IL-1β genes [5], [6] and with enzymatic inhibition of COX [4]. But, this study found that expression of COX-2 gene was not altered significantly by salicylate. Therefore, we suggested that the beneficial effects of *spirulina* or *C-PC* on tinnitus mainly via inhibiting mRNA expression of NR2B, TNF-α, IL-1β, and/or COX-2 genes.

Inflammation is associated with many neurodegenerative diseases, including Alzheimer’s disease [13], Parkinson’s disease [14], and many types of hearing impairment. For example, noise-induced cochlear damage [15], and cisplatin-induced ototoxicity [16]. Also, previous studies showed that TNF-α and IL-1β might interact with the NR [17], for example, in inflammatory hyperalgesia [18],and in spinal cord injury [19]. Recently, proinflammatory cytokines were linked to tinnitus [20]. We also found that the proinflammatory cytokines might lead to tinnitus directly or via modulating NR gene expression [5], [6].

S*pirulina* might be helpful for the neuroinflammatory and/or neurodegenerative diseases [11], [12]. *C-PC* has been shown to be a potent inhibitor of nicotinamide adenine dinucleotide phosphate (NADPH) oxidase [12]. On the other hand, the elevated cellular NADPH oxidase activity might contribute to their pathogenic impact, collaborating with increased iNOS activity to generate the cytotoxic oxidant peroxynitrite [12]. Wang et al. [21]reported that *Spirulina* can reduce the ischemia/reperfusion-induced apoptosis and cerebral infarction in mice with focal ischemia. Spirulina can also enhance striatal dopamine recovery and induce rapid, transient microglia activation after injury of the rat nigrostriatal dopamine system [22]. Our study group also found that *spirulina* could prevent memory dysfunction, reduce oxidative stress damage and augment catalase activity in senescence-accelerated mice [23]. Now, this study showed that *spirulina* or *C-PC* could significantly inhibit salicylate-induced over-expression of NR2B and proinflammatory genes.

As for the role of COX on tinnitus, Guitton et al. [4] hypothesized that the accumulation of arachidonic acid (AA) caused by inhibition of COX, could potentiate NR currents at the synapses between the inner hair cells and the dendrites of the cochlear spiral ganglion neuron in salicylate-induced tinnitus. Meanwhile, inhibition of COX could reduce inflammatory responses and prevent neural damages [11]. With these two contradictory or opposite sequelae, the point is that which pathways or mechanisms were more dominant for tinnitus production. In this study, we found that mRNA and protein expression levels of COX-2 gene were not inhibited significantly by salicylate injection, although COX could be inhibited at enzymatic level, but could be inhibited significantly by *spirulina* or *C-PC*. Thus, we supposed that anti-inflammation effect by COX inhibition could overcome the harmful effect of AA accumulation on NR activity, and was the dominant mechanism underlying the beneficial effect of *spirulina* or *C-PC* on salicylate-induced tinnitus.

Although this study showed significant results, there were still some weak points. According to our previous study, we found that the hearing thresholds elevated about 10 to 20 dB four days after salicylate injection, but elevated about 5–10 dB four days after salicylate injection plus spirulina water extract treatment. Thus, we had increased sound level of stimulation just for the salicylate-alone group, but not for salicylate plus spirulina and salicylate plus C-PC groups. Even though, we are not sure whether the sound level would need to be increased to maintain perceptual level for the salicylate group. And, we are also not sure whether the results would be violated by the increased sound levels in the salicylate group. Second, we could not document any potential changes in the expression levels of NR2B, TNF-alpha, IL-1beta, and COX-2 genes under spirulina or C-PC treatment in the absence of salicylate.

In conclusion, we suggested that *spirulina platensis* water extract and its active component (*C-PC*) could reduce salicylate-induced tinnitus. The beneficial role of *spirulina* mainly via inhibiting expression of NR, COX-2 and proinflammatory genes.

## Materials and Methods

### Animals

Three-month-old male SAMP8 mice (n = 96) weighing between 22.5 and 32.8 gm were randomly divided into four groups (n = 24 each) to receive saline (control group), salicylate treatment (tinnitus group), salicylate treatment plus *Spirulina platensis* water extract supplementation (*Spirulina* group), and salicylate treatment plus *C-PC* supplementation (*C-PC* group). The “control” and “tinnitus” groups were fed a normal diet, whereas the *Spirulina* group was fed a daily dietary supplement of *Spirulina platensis* water extract (1000 mg/kg body weight [BW]) and the *C-PC* group was fed *C-PC* (130 mg/kg BW) beginning on the first day of behavioral conditioning for tinnitus. Institutional Animal Care and Use Committee of Buddhist Dalin Tzu Chi General hospital had approved the protocol used in this study.

The *Spirulina platensis* water extract or *C-PC* were supplied by Far East Bio-Tec Co., Ltd. (Taipei, Taiwan). In brief, *Spirulina platensis* water extract was prepared as follows:

*Spirulina platensis* powder and pure water were mixed to form a suspension; the cells of *Spirulina platensis* in suspension were disrupted at a temperature lower than room temperature for 24 hours (patent pending) and centrifuged; the extract (supernatant) was collected and lyophilized. The lyophilized *Spirulina platensis* water extract contained 15–25% phycobiliproteins (C-phycocyanin and allophycocyanin), 35–45% polysaccharides, 10–20% proteins other than phycobiliproteins, 5–8% water, and 10–12% ash. The well-known active compounds in the extract are sulfated polysaccharides and phycobiliproteins.

### Behavioral Measurement of Tinnitus Score

All mice were trained in an active avoidance task, which was performed in a conditioning box with a climbing pole and a floor that could deliver an electric shock, according to the design of Guitton et al. [4] and Hwang et al. [5], [6].

### Conditioning to the Task

The conditioning paradigm consisted of 6 sessions per day (each lasting 15–20 min) with 10 trials per session performed for 5 days (day 1∼5). Inter-trial intervals were at least 1 minute. For each trial, the conditioning stimulus was a 50-dB sound pressure level (SPL) pure tone signal with a frequency of 10 kHz and a 3-second duration, and the unconditioned stimulus was a 3.7 mA electric foot-shock presented for up to 30 seconds, as described in Guitton’s protocol [4], by adjusting the voltage to the copper wire grid fixed to the floor.

The time between the conditioned stimulus and the unconditioned stimulus was 1 second. The mice would climb up the pole to reach a safe area after the coupled conditioned and unconditioned stimuli. Delivery of the electrical shocks was stopped by the experimenter when the animal climbed correctly. The “true-positive” score was the level of performance assessed by the number of times mice climbed correctly in response to sound. Mice were considered to be conditioned when the level of performance reached at least 80% in three consecutive sessions. Only conditioned mice were used the tinnitus experiments.

### Induction and Testing of Tinnitus

When conditioned, the mice rested for 1 day (day 6). Then, one session (10 trials) of an active avoidance task of was performed 2 hours after intraperitoneal injections of saline either alone or containing 300 mg/kg sodium salicylate (Sigma, St. Louis, MO) for 4 days (day 7∼10). To avoid changes attributable to hearing loss induced by salicylate (about 10–20 dB during 4 days of injections) [5], [6], the intensity of sound that elicited the behavioral responses was adjusted by increasing the sound intensity to 70 dB (SPL) for salicylate-treated group only. By doing so, the perceived level of sound in all mice in both groups was similar.

During testing, a sound of 3-second duration was given first in each trial, and the mice were observed for another 5 seconds to see whether they would perform the task correctly. If so (true-positive), the mice were put down on the floor for ongoing observation. If animals did not go to the safe area and stay >10 sec, an electrical shock was given by the experimenter to remind them to climb up. The mice were also put down on the floor for ongoing observation, if they stayed in the safe area >10 sec. Finally, the experimenter observed the total number (false-positive score or tinnitus score) of times that mice climbed during the inter-trial silent periods of 1 minute of 10 trials.

### Sample Isolation and RNA Extraction from the Cochlea and IC

The pairs of cochlea and IC were immediately dissected under a Zeiss stereomicroscope and stored at –80°C until use. Tissue was homogenized with a tissue homogenizer, and RNA was isolated using RNA-Bee isolation reagent (Friendswood, TX, USA) according to the manufacturer’s protocol. The RNA quality was assessed on the Agilent Bioanalyzer 2100 (Agilent Technologies, Palo Alto, CA, USA) and the ratio of absorbance measurements at 260 and 280 nm was obtained using a Nanodrop Spectrophotomer (NanoDrop, Wilmington, DE, USA).

### Reverse Transcription-polymerase Chain Reaction (RT-PCR)

cDNA was synthesized from total RNA by reverse transcription using a MasterAmp™ High Fidelity RT-PCR Kit (Epicentre Biotechnologies, Madison, WI, USA) in a P×2 Thermal cycler (Thermo Electron Corporation Bioscience Technologies Division, San Jose, CA, USA). For each RT reaction, a positive control (1 µg of total RNA) was included. RT was carried out at 37°C for 1 hour. For PCR amplification, 7.5 µL cDNA and primers were used according to the kit supplier’s instructions. To allow comparison of mRNA levels, we used β-actin as a loading control during quantitation of target mRNAs by RT-PCR. The primers were: NR2B-F, 5′-TCC GCC GAG AGT CCT CCG T-3′, NR2B-R, 5′-CTG CGT TGC CCT CGA TGT T-3′; TNF-α-F, 5′-CCCCTCAGCAAACCACCAAG-3′, TNF-α-R, 5′-CTTGGCAGATTGACCTCAGC-3′; IL-1β-F, 5′-GAGTGTGGATCCCAAGCAAT-3′, IL-1β-R, 5′-CTCAGTGCAGGCTATGACCA-3′; COX-2-F, 5′-CTG AAG CCC ACC CCA AAC A-3′, COX-2-R, 5′-AGT ATT CGC TCC TGG ACC CAA-3′; β-actin-F, 5′-CCACACCCGCCACCAGTTCG-3′, and β-actin-R, 5′-CCCATTCCCACCATCACACC-3′ (Protech-Taiwan, Taipei, Taiwan).

The thermal cycling conditions for PCR were as follows: 3 min initial set-up at 95°C; followed by 50 cycles, each of which consisted of 45 s of denaturation at 95°C, 45 s of annealing at 53°C, and 72 s of extension at 72°C for the TNF-α genes; of 45 s of denaturation at 95°C, 45 s of annealing at 52°C, and 72 s of extension at 72°C for the IL-1β gene; of 45 s of denaturation at 95°C, 45 s of annealing at 54°C, and 72 s of extension at 72°C for the NR2B gene; and of 45 s of denaturation at 95°C, 45 s of annealing at 50°C, and 72 s of extension at 72°C for the β-actin gene. A final 10 min extension at 72°C was performed for all the above genes.

### Quantitation of PCR Products

The DNA products were separated by the Mini Horizontal Electrophoresis System (MJ-105/MP-100; Major Science, Taipei, Taiwan) and analyzed using a E-Box-1000/26M Inspection Certificate & Analysis System (E-Box Spp-010 E-capt Software, Pharr, TX, USA). The expression levels of NR2B, TNF-α, IL-1β, and COX-2 genes are presented as relative ratios to that of β-actin.

### Western Blot Analysis

Equal amounts of the total protein in the IC were separated by 10% SDS–PAGE and transferred to nitrocellulose membranes, the membranes were soaked in blocking buffer (1% Bovine serum albumin). Proteins were detected using polyclonal antibodies against NR2B, TNF-α, IL-1β, or COX-2, and then visualized using goat-anti-rabbit or goat-anti-mouse IgG conjugated with peroxidase (HRP) as the HRP substrate. The expression level of above protein was presented as relative ratios in comparison to β-actin.

### Statistical Analysis

The data are presented as the mean ± standard deviation (SD), unless indicated otherwise. The expression levels of NR2B, TNF-α, IL-1β, or COX-2 genes were compared separately between four study groups by one-way ANOVA with post-hoc Bonferroni correction. All the above analyses were performed using the commercially available program STATA10, and p values <0.05 were considered statistically significant.
